# Experiences of accessing psychological therapies for self‐harm: A qualitative study of stakeholders' perspectives

**DOI:** 10.1111/papt.70089

**Published:** 2026-07-02

**Authors:** Tara Chauhan, Kamelia Harris, Isabel Adeyemi, Cameron Latham, Catherine Robinson, Stephen Kellett, Peter Taylor

**Affiliations:** ^1^ Division of Psychology and Mental Health, School of Health Sciences, Faculty of Biology, Medicine and Health Manchester Academic Health Sciences Centre, University of Manchester Manchester UK; ^2^ Greater Manchester Mental Health NHS Foundation Trust Manchester UK; ^3^ Division of Nursing, Midwifery and Social Work, School of Health Sciences, Faculty of Biology, Medicine and Health University of Manchester Manchester UK; ^4^ Rotherham Doncaster and South Humber NHS Foundation Trust Rotherham UK

**Keywords:** barriers, facilitators, psychological support, psychological therapy, qualitative interviews, self‐harm

## Abstract

**Objectives:**

While rates of self‐harm are rising in the United Kingdom, people who self‐harm can often struggle to access appropriate treatment. This study sought to explore adult stakeholders’ perspectives and experiences of the barriers to, and facilitators of, accessing support for self‐harm and also how services could be better tailored to meet needs.

**Design:**

A cross‐sectional qualitative study.

**Methods:**

Semi‐structured interviews were conducted remotely with 19 participants in the United Kingdom, including 10 individuals with lived experience of self‐harm and nine mental health professionals that worked with people who self‐harm. Reflexive thematic analysis was used to analyse the data. Normalisation Process Theory (NPT) was used as a guiding framework throughout the study.

**Results:**

Both stakeholder groups felt that services did not offer appropriate support; there were system‐level challenges and a sense of associated powerlessness. It was important to understand the person behind the behaviour, as a lack of understanding was reported to increase feelings of shame. Services that prioritise person‐centred care, expert‐by‐experience involvement and early intervention were valued. Professionals outlined the positives of brief interventions, while individuals who self‐harm held concerns about whether briefer interventions addressed complex difficulties.

**Conclusions:**

Findings highlight important organisational barriers and potential facilitators that individuals who self‐harm may face when accessing psychological interventions. Results prompt the need for inclusive and easily accessible services.

## INTRODUCTION

Self‐harm is defined as an intentional act of self‐injury or self‐poisoning, regardless of intention to die (National Institute for Health and Care Excellence [NICE], 2022). This can include self‐cutting and taking an overdose of medication. Less common methods include swallowing objects, insertion, burning, hanging and jumping from heights or in front of moving vehicles (NICE, 2022). Findings from the Adult Psychiatric Morbidity Survey (APMS) show that lifetime prevalence of self‐harm has risen from 2.4% in 2000 to approximately 10% of adults in 2023/2024, indicating a more than fourfold increase over time (Morris et al., 2025). Despite this, a significant proportion of individuals who self‑harm do not access formal support. Approximately 62.6% have no contact with services following self‐harm (McManus et al., 2019). Self‑harm is associated with a range of adverse long‑term outcomes, including an elevated risk of subsequent suicide attempts, ongoing psychological distress, and reduced life expectancy (Liu et al., 2022; Fazel et al., 2023). This highlights the need for rapid access to evidenced‐based and acceptable interventions for self‐harm.

Low rates of help‐seeking following self‐harm (e.g. Rowe et al., 2014) can be attributed to several factors. One barrier is the stigma of self‐harm, which intensifies feelings of shame, leading to secrecy and discouraging future help‐seeking (Long, 2018; Troya et al., 2019). Another barrier is perceptions that needs are not being met, feeling let down by services and difficulties in finding appropriate support (Wadman et al., 2018; Williams et al., 2020). A report by Samaritans, a UK mental health charity, highlighted a lack of effective interventions targeting self‐harm and the subsequent exclusion of individuals from services (Samaritans, 2020). Those who self‐harm were deemed ‘too risky’ for some services yet ‘not appropriate’ for others, resulting in individuals who self‐harm being passed between services (Samaritans, 2020). In the United Kingdom, General Practitioners (GPs) have expressed concerns about individuals consistently falling in the gap between the gap between National Health Service (NHS) Talking Therapies (a first line of psychological therapy support in the United Kingdom) and more specialist mental health services (Newbigging et al., 2018). These individuals are often excluded from NHS Talking Therapies yet may not meet the threshold for secondary care services, such as crisis and specialist personality disorder services (Steeg et al., 2024). Even individuals who are deemed eligible for support from services can face additional barriers. When accepted into a service, a lack of tailored interventions to specifically address self‐harm has been reported (Quinlivan, Gorman, Marks, et al., 2023). These challenges may exacerbate self‐harm behaviours and contribute to deteriorating mental health (Steeg et al., 2024).

UK national guidelines recommend cognitive behavioural therapy (CBT) interventions (NICE, 2022) for people who self‐harm. While there is some evidence that CBT and dialectical behavioural therapy (DBT) reduce self‐harm frequency, the quality of this evidence is low (Witt et al., 2021). A meta‐analysis of interventions for self‐harm found only small effects and there was no single consistently superior treatment (Fox et al., 2020). CBT may target depression or anxiety rather than self‐harm specifically, but self‐harm ideation can persist even when symptoms of depression reduce following CBT (Witt et al., 2021). DBT is an intensive, multiple‐component intervention. Due to the prolonged nature and intensity of DBT, it can be inappropriate for many young people who self‐harm (Witt et al., 2025). There is a need for facilitated access to appropriate, timely support (Saini et al., 2020). In addition, although psychodynamic interpersonal therapy (PIT) is not included within the NICE supported treatments, there is some evidence that it can be beneficial for self‐harm. For example, Guthrie et al. (2001) found that four sessions of PIT resulted in a reduction in self‐harm and suicidal ideation. However, evidence for PIT has been mixed, as PIT therapies were found to reduce self‐harm at a 6‐month follow‐up, but not a 12 month (Briggs et al., 2019). Brief therapies could increase timely access to psychological therapies for people who self‐harm. Brief relational psychotherapies that focus on self‐harm have been investigated recently and may hold promise in filling current gaps in services (Saini et al., 2021; Taylor et al., 2021, 2024, 2025). Cognitive analytic therapy (CAT) has been suggested as a time‐limited, relational therapy that may have value in helping people who self‐harm (Taylor et al., 2024, 2025), although further evaluation is required.

Implementing new interventions into existing services can be challenging (Grol & Grimshaw, 2003) and so understanding the service context is critical for understanding implementation barriers and facilitators. Qualitative research is particularly well suited for this purpose. For example, research focussed on self‐harm interventions designed for university students emphasised how concerns about privacy, and difficulties with accessing services were important barriers to consider for any new intervention (Cliffe & Stallard, 2023). To explore how brief relational therapies could fit within a community healthcare setting, it is important to understand the current treatment landscape. Gathering insights from multiple stakeholder groups on self‐harm experiences is recommended to inform the implementation process and to ensure the intervention aligns with service needs (Huddlestone et al., 2020). Different stakeholder groups may hold differing opinions and perspectives regarding self‐harm treatment. For example, individuals who self‐harm and clinicians working in this field appear to share common concerns (e.g. waiting times, restrictive service criteria) but also highlighted distinct issues (e.g. clinicians noted the fragmented nature of working between services as a cause of delays; Quinlivan, Gorman, Marks, et al., 2023; Quinlivan, Gorman, Monaghan, et al., 2023). These findings underscore the need to integrate diverse perspectives to inform more effective interventions.

Normalisation process theory (NPT; Murray et al., 2010) highlights the importance of understanding the current healthcare landscape and wider context in which interventions are placed, to assess how well a new approach would fit into a setting/system. NPT is useful in the early stages of intervention design to assess feasibility and to highlight the facilitators and barriers to the incorporation of an intervention into routine practice (Nilsen, 2020). This study aims to explore both the feasibility and real‐world application of a brief intervention for self‐harm; therefore, the theoretical model of NPT can help to explore what staff, services and organisations need to implement for an intervention to be successfully adopted into practice. As such, NPT can support with understanding how an intervention could work in real‐world settings, with consideration of the current context (May & Finch, 2009).

This study investigated the views of people with lived experience of self‐harm and mental health professionals who work with people who self‐harm in a healthcare setting using qualitative interviews. Compared to surveys, interviews enable deeper exploration of topics, adaptability to participant responses and rapport building (Burchett, 2014). This study had two aims: 1. to examine the perspectives of stakeholders for self‐harm interventions to understand the current service landscape of healthcare for adults who self‐harm, focussing on the barriers and facilitators to accessing psychological services and 2. to explore stakeholders' views about the usefulness of brief psychological therapies for self‐harm. This study was linked to a feasibility randomised controlled trial of a particular brief relational therapy, namely cognitive analytic therapy (CAT; Taylor et al., 2025) as a treatment for adults who self‐harm. However, despite this connection within this particular study, we investigated stakeholder views of brief psychological interventions more broadly, rather than narrowing our focus to CAT alone.

## METHODS

### Design

This was a qualitative study, using individual, semi‐structured interviews. This study received ethical approval from an NHS research ethics committee (Reference 22/NW/0317). The researcher was informed by a critical realist standpoint, which aligned with the aims of the study, to explore both participants' described experiences and the underlying structures influencing feasibility and implementation of brief interventions for self‐harm. From this perspective, we assume an external reality within which social and psychological processes operate but acknowledge that our view of this reality is affected by our position and context as researchers (Koopmans & Schiller, 2022). Normalisation Process Theory (NPT; Murray et al., 2010) was used as the guiding framework throughout the study. NPT informed the development of the interview topic guide, which focussed attention on participant views on the work required to implement new interventions. NPT also informed the analytical process, by focusing on factors that could support or hinder integration to clinical practice. The research team included a co‐investigator with lived experience of self‐harm who helped ensure this perspective was held in mind during the research. The RELATE (The Relational Approach to Self‐harm) trial Patient and Public Involvement and Engagement (PPIE) group were also consulted on developing the study aims and focus.

This study was part of the National Institute for Health Research (NIHR) funded study, Relational Approaches to Treating Self‐harm (RELATE; Taylor et al., 2024, 2025). The RELATE trial was a clinical trial investigating a talking therapy called Cognitive Analytic Therapy (CAT) as a treatment for adults who self‐harm. This qualitative study had the objective of better understanding the landscape of services for self‐harm within the NHS, and as such intentionally adopted a broader perspective than the RELATE trial, not focussing specifically on CAT as an approach. Individuals connected to the RELATE trial were eligible to participate in this study, for example, trial therapists and members of the PPIE advisory group. However, most participants were recruited externally, which hoped to capture a broad range of perspectives.

### Participants

Four different stakeholder groups were recruited: (i) individuals with lived experience of self‐harm, (ii) health professionals working with individuals who self‐harm, (iii) parents/carers of individuals who self‐harm and (iv) professionals working for third‐sector organisations, supporting individuals who self‐harm. All participants had to be aged 18 years or over. Although parents/carers were in the planned sample, no participants from this group were successfully recruited. Additional inclusion criteria are detailed in Table 1.

**TABLE 1:** Inclusion and exclusion criteria.

Participant group	Inclusion criteria	Exclusion criteria
Individuals with lived experience of self‐harm	Aged 18 years or over Previously accessed or tried to access psychological support services Direct lived experience of self‐harm, currently or historically	Moderate‐to‐severe learning disability or cognitive impairments. This is due to such individuals not being eligible for community psychological services, which is the focus of this research Individuals who do not speak English to a level that impacts their ability to participate in the interview
Individuals with lived experience caring for/supporting individuals who self‐harm	People who are currently caring for or who have previously cared for an individual who self‐harms	Individuals who do not speak English to a level that impacts their ability to participate in the interview
NHS health professionals who work with people who self‐harm	Clinical staff currently working in NHS or third sector organisation community settings and involved in the care of individuals who self‐harm (e.g. GP, Health Care Assistants, Psychologists, Nurses, Psychiatrists, support workers)	People working for the NHS who are not involved directly in the provision of care/support (e.g. domestic staff, administrators)
Professionals working for third sector organisations who support individuals who self‐harm	Staff currently working in third‐sector organisations involved in supporting individuals who self‐harm	People who work for third‐sector organisations who are not directly involved in the care or support of individuals who self‐harm

A target sample of 20 stakeholders was sought, guided by the principles of information power (Malterud et al., 2016). Following these principles, the researcher placed the study along a continuum focusing on the study aim, theoretical background, analysis strategy, quality of dialogue and sample specificity, to determine an optimal sample. For example, the study was judged to have a broad aim, and the quality of the interview dialogue was predicted to be strong, with rich data.

### Procedure

The study was advertised to individuals with lived experience by contacting self‐harm charities who posted the advert on their private social media groups (e.g. Facebook) and circulated the study via email. The advert was also circulated to the PPIE group of the RELATE trial (Taylor et al., 2024, 2025), comprising people with lived experience of self‐harm. Additionally, the study was advertised to healthcare professionals by contacting local NHS mental health service leads and emailing the clinicians involved in the RELATE trial.

Individuals who wished to take part were invited to attend a meeting with the researcher via video call or face‐to‐face. Participant information was sent via email before the first meeting with the researcher. All participants opted to be interviewed via Microsoft Teams. Informed consent was taken at the start of the research meeting. Participants then completed a demographic form, in which the researcher read out each question. The semi‐structured interview was audio‐recorded via an encrypted audio‐recording device. People with lived experience received a £15 shopping e‐voucher via email for participating. Health professionals did not receive an e‐voucher. Interviews lasted between 44 min and 60 min (median = 49 min 47 s).

### Materials

Separate demographic forms were used for individuals with lived experience and health professionals. Both participant groups were asked about their age, gender identity, sex assigned at birth and ethnicity. Information about the type and length of support received from services was gathered from individuals with lived experience. Professionals provided information about their level of education, the kind of therapy they had delivered (if any), how long they had worked in their current role, and how they would describe their experience of working with individuals who self‐harm (i.e. rarely, occasional clinical contact or frequent clinical contact).

Separate interview topic guides were developed for each stakeholder group. The study aims were used to guide the development of questions, and we aimed to create open questions that would still elicit content relevant to the study aims (e.g. ‘What do you think are the challenges facing adults who self‐harm who are seeking therapy’). The topic guides were developed and refined with the wider research team, following consultations with a PPIE group and co‐investigator with lived experience of self‐harm. This consultation shaped the interview schedule through prompting the researcher to consider the wording of questions, with recommendations to start with broader questions, to help to build rapport and avoid pathologising language. The NPT (Murray et al., 2010) was used to guide the questions around barriers to and facilitators of accessing therapy, service uptake, and staff appraisal. The key areas of NPT and examples of interview questions linked to these areas are outlined in Table 2. Both topic guides were used if a participant belonged to more than one stakeholder group. Participants were encouraged to freely share their views and experiences. Both topic guides began with broad, open‐ended questions. Individuals with lived experience were asked about their experiences of seeking support, while professionals were asked about their experiences of providing support for people who self‐harm. Both groups were asked about their thoughts on brief psychological interventions, potential missed opportunities in services for adults who self‐harm and key considerations for effective therapy.

**TABLE 2:** Key areas of NPT.

NPT component	Definition	Example interview question
Coherence	The meaning and sense‐making by the participant	What is your perception of the current provision/availability of therapies for adults who self‐harm?
2 Cognitive participation	Commitment and engagement	How would you feel if you were seeking help and were offered a brief psychological therapy to help with self‐harm?
3 Collective action	The work people do to enact the implementation	How might this approach sit alongside other approaches for self‐harm? Do you have any hopes or concerns about this?
4 Reflexive monitoring	Participants reflect on/appraise the intervention	How do you think other members of your team/service/setting would view this approach?

### Analysis

Transcribed interview data were analysed by the first author using reflexive thematic analysis (Braun & Clarke, 2019). During transcription, identifiable information was removed. NVivo 12 qualitative software was used to manage the anonymised transcripts (Lumivero, 2017). A critical realist perspective provided the researcher with a lens to understand how these experiences could be shaped by the researcher's and participants' broader social, cultural and structural contexts (Zhang, 2023). The analysis used a primarily inductive approach, whereby an assumed reality existed within the data, allowing the researcher to generate insights directly from participant experiences (Braun & Clarke, 2013). However, a deductive process was then also adopted, using NPT to guide interpretation. NPT was used to inform the analytic lens; the NPT constructs helped to interpret patterns in the data and refine developing themes, while ensuring that themes were grounded in participant accounts. NPT therefore supported the organisation, interpretation and coherence of the final themes. The study aims informed the interview schedule and so interviews naturally generated content relevant to the aims, but the aims were also held in mind during analysis, so that codes and shared concepts that spoke to the study aims were sought.

The six stages of thematic analysis, as outlined by Braun and Clarke (2019), were undertaken by the first author. Data were read and re‐read several times to achieve familiarisation. A hybrid approach integrated both semantic and latent coding to provide a more nuanced understanding of participant experiences by capturing both explicitly expressed meaning and deeper, more implicit meaning (Braun & Clarke, 2021). The initial themes were discussed with the wider research team and a mental health consultant with lived experience of self‐harm. The final themes were refined and named through discussion with the research team members. Data from the different stakeholder groups was analysed together as one corpus, facilitating the identification of common themes, but the researcher remained mindful of any points of disagreement or tension between members of different groups. Group‐specific points were highlighted as variations within the themes and whereby certain ideas were especially relevant to one group, such as opinions on brief therapy, this is reported clearly in the results.

### Reflexivity

The first author maintained a reflexive journal to document reflections on methodological decisions, participant interviews and data analysis (Malterud, 2001). They considered how their identity as a heterosexual woman of mixed ethnic background and a Trainee Clinical Psychologist in the NHS might have influenced the research. Acknowledging their prior experiences of self‐harm support challenges, both professionally and personally, the author reflected on assumptions regarding gaps in service provision. The idea of multiple realities and critically reflexive questioning helped the author to be mindful of the impact of their assumptions on the analytic process and remain curious and open to new understandings (Cunliffe, 2016). The reflexive journal helped record the ongoing research process, including practices, assumptions made and experiences of supervision.

## RESULTS

### Participant characteristics

Nineteen participants were interviewed. Two participants belonged to more than one stakeholder group, one healthcare professional disclosed a history of self‐harm, and one of the individuals with lived experience of self‐harm also had a paid role at a third‐sector organisation. Due to the small number in the third sector stakeholder group (*n* = 1), they are referred to as belonging to the lived experience stakeholder group for the purposes of reporting. However, no parents or carers of individuals who self‐harm expressed an interest in taking part. Ten individuals with lived experience were recruited. Two of the participants with lived experience of self‐harm were recruited from the RELATE trial PPIE group. Participants with lived experience were aged between 22 and 60 years (M = 37.9, SD = 12.21). The number of years participants reported receiving support for their self‐harm ranged from 1 to 32 years (M = 13.5, SD = 11.21). Table 3 provides further details on participants with lived experience characteristics. Nine healthcare professionals were recruited. Two were trial therapists working on the RELATE trial. The healthcare professionals' ages ranged from 22 to 46 years (M = 37.44, SD = 6.58), and they had worked in their current role for an average of 2.17 years. One healthcare professional also disclosed a history of self‐harm. Table 3 provides further details on the characteristics of healthcare professionals.

**TABLE 3:** Participants' demographic information.

Demographic	Lived experience group, *N* (%)	Practitioner group, *N* (%)
Gender		
Female	8 (80)	7 (78)
Male	1 (10)	2 (22)
Non‐binary	1 (10)	0 (0)
Ethnicity		
White‐British	5 (50)	7 (78)
Asian‐British	3 (30)	1 (11)
Indian	2 (20)	0 (0)
Other	0 (0)	1 (11)
Services received support from		
GP Clinic	3 (30)	–
Crisis Team	2 (20)	
Adult Community Mental Health	7 (70)	
NHS talking therapies (IAPT)	2 (20)	
Specialist Psychological Services	1 (10)	
Acute Psychiatric Care	1 (10)	
Private Counselling	3 (30)	
Charities	5 (50)	
Accessed therapy for self‐harm before		–
No	1 (10)	
Yes	9 (90)	
Type of therapy received		
Cognitive behavioural therapy	5 (50)	
Cognitive analytical therapy	2 (20)	
Compassion focussed therapy	2 (20)	
Dialectical behavioural therapy	3 (30)	
EMDR	1 (10)	
Private psychotherapy	3 (30)	
Group therapy	1 (10)	
Worked for a third‐sector organisation		
Yes	1 (10)	
No	9 (90)	
Highest professional qualification	–	
Doctorate in clinical psychology		6 (67)
Master of Science/Master of Art		2 (22)
Diploma or equivalent		1 (11)
Professional role	–	
Clinical Psychologist		4 (44)
Consultant Clinical Psychologist		2 (22)
Mental Health Practitioner		1 (11)
Psychological Well‐Being Practitioner		1 (11)
Senior Psychological Therapist		1 (11)
Description of working with self‐harm	**–**	
Frequent Clinical Contact		8 (89)
Occasional Clinical Contact		1 (11)

### Key findings

Three themes emerged: (i) feeling powerless within the system, (ii) understanding the person behind self‐harm and (iii) what could self‐harm support look like? A thematic map (see Figure 1) illustrates the connections and interactions between the themes. While there was substantial agreement between stakeholder groups, some sub‐themes were more relevant to one group. For example, the importance of involving self‐harm experts by experience seemed more pertinent to the individuals with lived experience. There was some divergence of views within the data, whereby professionals saw the advantages of a brief, time‐limited therapy. This contrasted with the opinions of some individuals with lived experience who thought such therapy would not meet their needs. Within quotes, individuals with lived experience have been referred to as ‘LE’, and health care professionals have been referred to as ‘HC’, followed by a unique identifier. These results all link into the constructs of NPT in various ways, please see table 4 for more information.

**FIGURE 1:**
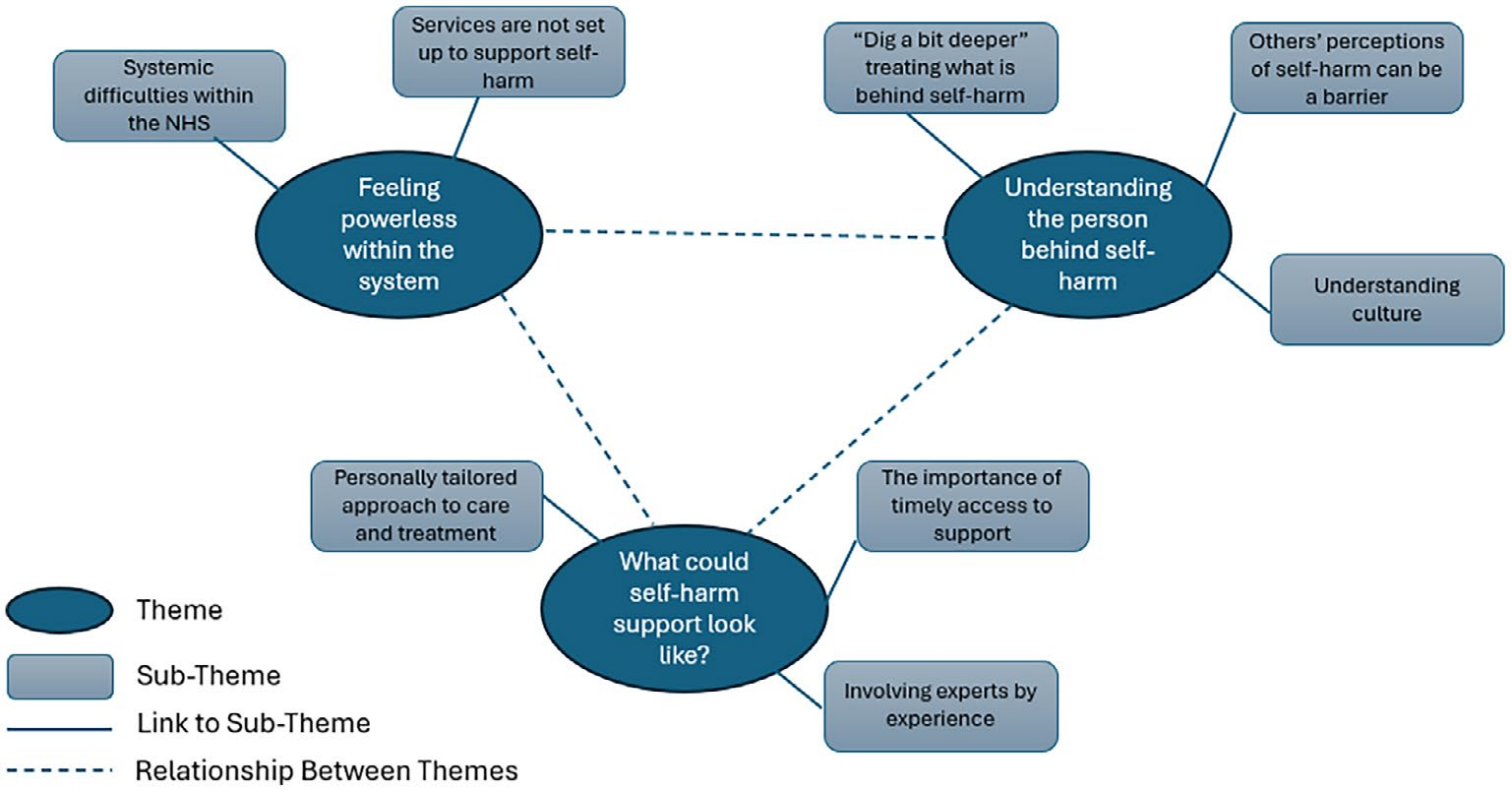
Structure of the themes and subthemes.

### Theme 1: Feeling powerless within the system

Both stakeholder groups noted that individuals who self‐harmed faced significant systemic and service‐level barriers to accessing appropriate care. Systemic funding constraints and inadequate service structures were perceived to lead to exclusionary practices, such as individuals with lived experience of self‐harm being told that they did not meet the criteria for the service, in which individuals who self‐harmed were unable to access needed support. Both individuals with lived experience and professionals expressed frustration and a shared sense of powerlessness, whereby they felt unable to initiate meaningful change within a rigid healthcare system.

#### Systemic difficulties

Participants were aware of the system‐level barriers to accessing support for self‐harm, which included a persistent lack of available resources within the NHS (e.g. not enough professionals to accommodate the demand for services) and long waiting times. These barriers were attributed to the underfunding of healthcare services:

To me, the NHS is just broken, and all people are doing is…they're just putting little sticking plasters on massive, big wounds all the time because they haven't got the resources, the time, the people even to do the actual work that needs to be done. (LE2)

Both stakeholder groups commonly highlighted long waiting times for mental health support. They described how prolonged waits for individuals who self‐harm could lead to a deterioration in their mental health, resulting in a heightened risk of further self‐harm. Despite an awareness of the consequences of these long waiting times, both groups shared a sense of powerlessness to make changes and overcome the systemic barriers within the NHS, such as funding shortages, resource limitations and high demand for mental health support.

Further harm is going to be done to those people individually, systemically, relationally during those waiting periods. I think the really frightening thing is how little power and scope we have, I think, to improve that. (HC2)

This powerlessness created a cycle of frustration, followed by disengagement from services for individuals who self‐harm. The long waiting time, sometimes 3 years, exacerbated existing negative perceptions of self, such as undeserving of support. Additionally, participants with lived experience described how they might have ceased seeking support after long waits, repeated rejections from services or feeling that help was only available at times of crisis, for example, if there was an immediate risk of suicide.Both clinicians and individuals with lived experience of self‐harm expressed a sense of powerlessness in navigating service barriers and implementing changes, informing lower levels of collective action, in line with NPT.

#### Services are not set up for people who self‐harm

A sense of powerlessness also arose from individuals with lived experience and healthcare professionals who described that services often were not set up for people who self‐harm. For example, individuals who self‐harm described barriers they experienced, such as being deemed ‘too complex’ and ‘risky’ for particular services or not severe enough for other services. Professionals highlighted that service policies meant that they were unable to manage or contain the risks associated with self‐harm. Both stakeholder groups described that services would not provide support to individuals who actively self‐harmed. Professionals explained that individuals were told to ‘manage their own safety’ before the service accepts them. Individuals with lived experience reported that when they were offered therapy, it did not focus on self‐harm specifically, and instead they were provided therapy for trauma or depression, which often ‘ignored’ self‐harm:

If you're actively self‐harming or if you've actively got suicide ideation…a lot of services won't touch you, they'll pass the buck on and say, we don't deal with that. (LE2)

Both stakeholder groups described a feeling that services and clinicians could be ‘scared of’ self‐harm and, therefore, reluctant to provide support to individuals due to their perceived risk. This could lead to individuals being ‘rejected’ from services without being given alternative sources of support. The barriers set up by services that exclude people who self‐harm could result in a reluctance to disclose or have any meaningful discussions with professionals about such experiences. Individuals felt compelled to distort the truth about their own experiences to fit into the services' eligibility criteria and receive any professional support. Even after receiving support, they often found it challenging to open up about self‐harm experiences during therapy, fearing negative consequences such as discharge from the service or professionals ‘overreacting’ to risk:

They can't say that they self‐harm because, if they said they self‐harm, the therapy that they're having would be withdrawn. […] So, like, imagine having therapy and not actually being able to be honest. (LE2)

Healthcare professionals explained that individuals with lived experience were often told that they were too risky for NHS Talking Therapies and were advised they had been referred to secondary care (specialist crisis or complex needs services). However, they may then be told that they do not meet the criteria for secondary care or can face another long wait for this support. In both cases, individuals explained that they were often passed between services and left with no or fragmented support.

Both participant groups identified a gap in the current therapeutic approaches offered by services, which did not specifically focus on self‐harm. Individuals with lived experience commonly described that their self‐harm was ‘never considered’ in therapy. The exclusion of individuals' self‐harm experiences could result in feelings of shame and discourage help‐seeking. In addition, the gaps within NHS services for those who self‐harm could make people compelled to seek private sources of support to meet their needs. Charities were highlighted as vital ‘lifelines’, providing a safe, non‐judgmental therapeutic space where no other avenues for self‐harm support were available.

### Theme 2: Understanding the person behind self‐harm

Individuals with lived experience and healthcare professionals described the importance of understanding the needs of individuals beyond their self‐harm. They reported that support for self‐harm can be behaviourally focussed, which targets getting ‘rid’ of the self‐harm behaviour. Instead, the importance of getting to the ‘root’ of the problem was highlighted. This included the importance of looking beyond the self‐harm behaviour to explore the root causes and understand ‘why’, considering wider contextual factors, such as culture and exploring previous traumatic or difficult life events that may have triggered self‐harm. A few professionals explained that when clinicians ‘panic’ about self‐harm, fearing the risks associated with self‐harm, they miss important opportunities to understand and work with self‐harm. When professionals do not explore these underlying factors, it can exacerbate the stigma surrounding self‐harm and create additional barriers to accessing support.

#### ‘Dig a bit deeper’—Understanding self‐harm

Both stakeholder groups commonly expressed the difficulties of addressing the complexities of self‐harm within therapy. Self‐harm was described as ‘the tip of the iceberg’, whereby the underlying reasons behind self‐harm required further exploration from clinicians. It was felt that self‐harm could be a ‘symptom’ of other factors: ‘This is how I'm coping, and yes, there's a little bit of self‐harm going on, but let's work together to figure it out and dig a bit deeper’. (LE10).

For example, it was described how self‐harm could be a result of other problems or a manifestation of more profound underlying difficulties. Although the importance of getting to the ‘why’ behind self‐harm was frequently reported by both stakeholder groups, this did not reflect the experiences shared by individuals with lived experience. According to participants with lived experience, both current approaches to self‐harm and professionals who provide support were not interested in the underlying reasons behind self‐harm and often ignored these deeper issues. This could lead to additional barriers to support, whereby individuals reported shutting down to further engagement: ‘They wasn't interested in the whys. And for me, I think that became a big barrier because…I then started closing off because I was like, what's the point’. (LE6).

According to both stakeholder groups, behavioural approaches to self‐harm, such as DBT and CBT, do not get to the ‘root’ of the problem. DBT was described as a ‘sticking plaster’ approach, with limited helpfulness in the long term, in which individuals are left requiring further support.

#### Others' perceptions of self‐harm

The judgements that individuals who self‐harm made towards themselves and faced from others were identified as a barrier to receiving support from both stakeholder groups. Notably, both stakeholder groups described how individuals who self‐harm could already have a negative view of themselves for their self‐harm; therefore, when others judge them, this could exacerbate how they were already feeling and could have a more significant impact:

There's nobody judges me more than I judge myself, I'm my biggest judge, and I'm probably my harshest judge […] when somebody else judges me, it just compounds on that, and then it makes me feel worse than I did before I before I went there. (LE10)

The way that services respond to individuals who self‐harm could feed into the already negative view that they may hold of themselves. Individuals with lived experience described how they could feel high levels of shame surrounding their self‐harm; this could then make it difficult to push or ask for support. Therefore, when they are told by services that there is no support, that they are too risky, or not risky enough for support, or if they sense judgement from services, this could result in individuals feeling higher levels of shame and unable to push for further help or advocate for themselves.

Because of that shame, I tend not to push for help. So … when they are like, oh well, there's nothing we can do right now, call back another time. I won't call back, because I already feel like a burden and like I'm ashamed of it. (LE8)

From a clinician's perspective, this sense of shame may have come from individuals who self‐harm experiences when previously trying to access support. Therefore, there appears to be a vicious cycle in which feelings of shame regarding self‐harm could be a barrier for individuals asking for help. When they do ask for help, they could be shamed by that experience, which could prevent individuals from feeling able to seek support again.

Both stakeholder groups described that individuals could be given medical labels such as ‘Personality Disorder’ by professionals that do not match their experiences; these labels were often viewed as hindering access to further support. As mentioned in the earlier theme, *services are not set up to support self‐harm*; it was highlighted how individuals could distort their experiences to fit in with what services want. This theme highlights how, in addition to this, services could distort individuals' experiences so they, too, ‘fit’ into a set criterion. Some participants described that being given labels would facilitate and allow for support from specific services. However, this contrasted with other participants' views that inappropriate labels could feed into negative perceptions they may hold of themselves and create further barriers. In both cases, individuals are being placed, either by themselves or the wider professionals around them, into inappropriate boxes:

I somehow ended up with a personality disorder diagnosis which makes absolutely no sense to anybody involved in my care […] the second that I had that on my records I noticed that the support really withdrew. (LE3)

#### Understanding culture

Individuals with lived experience from a non‐White‐British background discussed that there could be cultural barriers to accessing support, such as stigma surrounding mental health and self‐harm within their culture, differing attitudes towards seeking help and language barriers that could hinder family understanding and support. Individuals revealed cultural differences in understanding of mental health, which may not align with Western medical models of mental health and help‐seeking. These differences in understanding could perpetuate feelings of shame regarding seeking help for self‐harm, which could create further barriers to help‐seeking. A need for an increase in cultural awareness from services was shared:

It almost felt wrong, the culture within the household, to seek help, to get help for self‐harm. […] Maybe if services had more of a cultural awareness as well of, kind of, the shame someone might be bringing, that could help. (LE9)

One individual described questioning the reasons behind being passed between or rejected from services when seeking support for self‐harm. This highlights the importance of how the political context and an individual's wider experiences of racism could create mistrust towards services and a reluctance to seek further help:

When I go to [location], I'm aware that I'm going into areas where there are no brown faces and where there might be, kind of like, opinions. And I know that in [redacted] City Centre there have been far right marches […] when I'd finished speaking on the phone for [redacted] service today I did wonder is this actually a race issue, not a oh no, we can't deal with your issues. (LE2)

### Theme 3: What could self‐harm support look like?

This theme describes participants' reflections on how self‐harm services could be improved. Healthcare professionals and individuals with lived experience agreed about the importance of early intervention for self‐harm. However, while healthcare professionals unanimously highlighted the benefits of brief therapy, individuals with lived experience presented a more nuanced perspective, with some expressing concerns about the ‘artificial’ length of brief interventions. For example, individuals with lived experience described concerns that therapy would end abruptly before they were ready, explaining that it may not allow for time to develop a therapeutic relationship or address their underlying and sometimes complex difficulties. However, some individuals with lived experience suggested that such approaches could be beneficial for those initially seeking support for self‐harm as an early intervention before they felt their difficulties became more complex to address. Despite these differences, both groups emphasised the need for more diverse options and choices for individuals who self‐harm, which differed from the traditional options of CBT or DBT.

#### The importance of timing access to support

Opinions about the benefits of brief therapy for self‐harm diverged between individuals with lived experience and professionals. For example, people with lived experience felt that a time‐limited therapy would not give opportunities to explore the complex reasons behind self‐harm in‐depth, especially if those experiences have been occurring for a long time, and to establish a good working relationship with a therapist, which is conducive to openly discussing experiences of self‐harm:

I know that it takes me a long time to trust people and open up with them. And so, I know that if they offered me eight weeks, I'd be like, no. That's going to make it worse. (LE8).

This contrasted the views of many healthcare professionals, who emphasised the usefulness of brief interventions and highlighted how interventions should be needs‐led, whereby not every person requires longer‐term therapy. In addition, having a clear boundary to the ending of therapy was suggested as helpful for people who struggle with endings. Despite this, both groups acknowledged the importance of early intervention and many individuals with lived experience reported that a brief approach focused on self‐harm would have been beneficial as an intervention when they initially sought help, which may have prevented difficulties from worsening:

If that could be offered initially to people, say, when they were presenting at A&E or speaking to the GP for the first time about self‐harm, if that was something that could be offered early on as, like, an initial response…I think that could help. (LE4)

#### Involving experts by experience

The value of involving experts by experience (EbEs) in service design and delivery was mentioned frequently by individuals with lived experience. Individuals with lived experience of self‐harm have a unique awareness and understanding. It was therefore suggested that EbEs should be involved in training clinicians, which could increase understanding and empathy and bridge the gap between clinical knowledge and personal experience. Furthermore, it was suggested that peer support could provide a safe, non‐judgmental space to disclose self‐harm experiences, which could subsequently reduce feelings of shame and stigma:

Because when you're dealing with somebody who's actually been through what you're going through and they understand where you are, you don't feel like you've got somebody who you're just another number to. (L6).

This was mentioned less often by healthcare professionals; however, one professional acknowledged that not involving EbEs in service provision represented a missed opportunity. They suggested that involving people with lived experience in research on self‐harm to increase the evidence base could help tailor service design and delivery to better meet individuals' needs. These findings map onto areas of the NPT, such as coherence, cognitive participation, collective action and reflexive monitoring (Murray et al., 2010).

#### Person‐centred care

Individuals with lived experience felt that person‐centred care, tailored to individuals' needs was paramount. Similarly, clinicians emphasised the importance of tailoring formulation and understanding of experiences to an individual's needs. However, individuals with lived experience expressed that manualised or ‘textbook’ therapies, such as dialectical behaviour therapy (DBT), did not optimally address individuals' needs. Being offered a choice of support and understanding whether that support is right for the person with lived experience was perceived to be lacking in services. Similar to the previous theme, which outlined the importance of understanding a person's self‐harm, individuals with lived experience reported that when therapies that focus on understanding the person beyond their self‐harm behaviour, they were perceived as more person‐centred. In addition, individuals' awareness of the available therapies and what they entail, and choice of therapy would mean that support was person‐centred and individualised:

If there are other options available on the NHS they're not really talked about. […] Nobody gives you anything beyond CBT that I'm aware of…like, you're going to do this therapy and it's going to work, without any actual understanding of how it works or whether it's the right thing. (LE3).

Although individuals with lived experience had certain reservations about the usefulness of a brief therapy for self‐harm, they agreed that it would nevertheless ultimately increase choice for individuals who self‐harm. This resonated with clinicians' views:

I think clinicians would welcome it [a brief therapy], to be honest, the more options they've got the easier it is to match someone's needs to something within the service […] I think service users respond well to knowing there's something that's a bit more tailored to what they need. (HC6).

## DISCUSSION

This study examined the perspectives of stakeholders to understand the current service landscape of health care for adults who self‐harm, focussing on the barriers and facilitators of access and exploring views about the usefulness of a brief psychological therapy. To our knowledge, this is the first study to investigate both individuals' who self‐harm and professionals' working with self‐harm views on the benefits of brief, time‐limited therapy. Results indicated that both stakeholder groups felt powerless within the care system, highlighting systemic barriers, such as long waiting times and exclusion from services due to risk concerns and case complexity. Participants emphasised the importance of therapies focussing on understanding the reasons behind self‐harm, which included culturally‐ and context‐sensitive considerations. For example, participants described the shame and stigma which could impact their help‐seeking and the impact of experiencing racism in their wider environment. Participants also stressed the need for person‐centred approaches, the need for wider patient choice and therapy options specifically tailored to people who self‐harm. The importance of early intervention was highlighted. Specifically, brief therapies were seen as an appropriate early intervention that could increase patient choice. Including EbEs in services to support the training of clinicians and service development was recommended. These findings map onto areas of the NPT, such as coherence, cognitive participation, collective action and reflexive monitoring (Murray et al., 2010).

This study highlighted significant barriers, aligning with previous research by Quinlivan, Gorman, Marks, et al. (2023) and Samaritans (2020). Participants reported long waits for services and frequently being excluded from and passed from service to service, often without being informed of alternative support options. This led to individuals not receiving any support/care or feeling pressure to conform to specific service criteria to access support. These findings contribute to research which highlights how negative responses to the disclosure of self‐harm can become a barrier to further disclosure (Rosenrot & Lewis, 2018). Therefore, the above negative experiences of services could further impede the disclosure of self‐harm to professionals. Charities were noted as vital in filling the gaps of service provision, which is consistent with previous research outlining the importance of the voluntary sector for people in crisis (Newbigging et al., 2017). Both stakeholder groups' awareness of the shortcomings in the current provision of therapy and identification of the gaps within services related to the idea of coherence within NPT, suggesting a coherent perceived need for new interventions or service changes (Murray et al., 2010). Previous research has suggested that clinicians working in non‐managerial roles within the NHS can feel powerless to influence organisational/systemic level change (Ng, 2022). Similarly, in this study, both clinicians and individuals with lived experience of self‐harm expressed a sense of powerlessness in navigating service barriers and implementing changes, informing lower levels of collective action.

A need for greater choice of interventions and tailored individual support is documented in previous research (Lindgren et al., 2018; Quinlivan, Gorman, Marks, et al., 2023). In this study, frustrations centred on the inadequacy of existing therapies, which were described as overly behaviourally focussed, aiming to reduce acts of self‐harm. Therapy acted as a ‘sticking plaster’ rather than understanding the underlying reasons for self‐harm and addressing the underlying difficulties. Current therapy options were reported to focus on specific mental health difficulties (e.g. depression, anxiety) and ignored self‐harm entirely. This supports the findings from a recent meta‐synthesis showing that recognising and understanding the person beyond their self‐harm behaviour was key in effectively supporting people who self‐harm (Haw et al., 2023).

While clinicians viewed brief therapy as beneficial, individuals with lived experience expressed reservations about having fewer sessions, due to concerns about insufficient time to open up to a therapist and build a good working relationship. Due to resource constraints within the NHS, time‐limited treatments are widely used (Iqbal et al., 2021). But the end of brief psychological therapy can be difficult and brings up complex emotions, particularly when the ending is not discussed or planned (Knox et al., 2011). This study highlights the need for careful consideration of therapy ending when implementing brief interventions. Despite these conflicting views regarding brief interventions, both groups acknowledged the potential advantages, specifically in the early stages of self‐harm experiences, and the importance of increased early intervention options, person‐centred care, and reduced waiting times. The importance of early intervention in self‐harm support is well documented (Knox et al., 2011). Through their analysis of the costs and benefits of brief interventions, both stakeholder groups demonstrated reflexive monitoring in line with NPT, showing high cognitive participation and willingness to invest in the intervention (Murray et al., 2010).

An important contribution of this study is that it identified the cultural factors that could shape experiences of self‐harm and help‐seeking. Participants from an ethnically minoritised background shared unique cultural barriers such as stigma surrounding mental health, differing cultural norms surrounding help‐seeking, and concerns about discrimination or racism within services. These results highlight how an individual's cultural context can intersect with shame, trust of services and disclosure of self‐harm. This aligns with recent research which highlights the importance of culturally sensitive approaches for mental health care and the importance of considering socio‐cultural contexts within interventions for self‐harm (Venkatesan, 2024).

Previous studies have investigated the barriers and facilitators to self‐harm support qualitatively with clinicians and individuals with lived experience separately (Quinlivan, Gorman, Marks, et al., 2023; Quinlivan, Gorman, Monaghan, et al., 2023). However, by interviewing multiple stakeholder groups, the researcher could explore whether first‐hand lived experience accounts of individuals who self‐harmed and their experiences of trying to access support were similar to those of the clinicians delivering support. Diverging and converging experiences of individuals who self‐harmed and professionals supporting people who self‐harm were examined.

### Strengths and limitations

The homogeneity of the participant characteristics was a limitation. Some groups were underrepresented in the sample, and so their views were not captured. For example, the clinicians interviewed were predominantly white, cisgender women recruited from two NHS trusts in the North‐West of England. Additionally, the individuals with lived experience were predominantly female, from a White British or Asian British background, and were recruited mostly from self‐harm charities and support groups. Future research should explore the experiences of accessing support for self‐harm from individuals and clinicians from various ethnic, gender identity and sexual orientation backgrounds and different marginalised identities and geographical locations to fully capture the experiences of people who self‐harm. Two clinicians who were therapists on the RELATE trial and two individuals with lived experience who were involved in the trial's PPIE group were included in this study. Interviewing these individuals was valuable, given their experience and knowledge of self‐harm service provision and the needs of those who self‐harm. These participants were not specifically asked about the RELATE trial or the therapy tested in the trial, as the focus of this study was understanding the current self‐harm service landscape more broadly. Nonetheless, those participants' involvement in the RELATE trial may have impacted their views and attitudes towards brief psychological therapy for self‐harm. However, this study also captured the views of participants not involved in the RELATE trial, and there did not appear to be differences in views regarding support for self‐harm and brief therapy for self‐harm.

A further limitation of this study was the absence of parents/carers of individuals who self‐harm from the sample, despite plans to recruit this stakeholder group. No individuals who identified as carers for individuals who self‐harmed responded to adverts. This may be because of the focus here on services for adults who self‐harm (the researcher contacted mental health and self‐harm charities which predominantly supported adults), rather than young people, where parents and carers may be more involved. Future research could recruit parents and carers of people who self‐harm by considering a broader range of recruitment locations including services working with young people. We do not have information on how many people viewed adverts for the study but decided not to take part, and we did not have ethical approval to retain information on those who contacted the research team but ultimately decided not to take part. As such, it is hard to ascertain how representative our sample is of the wider pool of potential participants.

COREQ is widely used in qualitative health research and can support comprehensive reporting of interview‐based studies (Tong et al., 2007). Although the reporting of this study followed core principles of qualitative rigour, such as clear methodology, reflexivity, the transparency of the analytic process and grounding the themes in the data, the researcher not explicitly using COREQ could be considered a limitation. However, there are mixed views in qualitative research, whereby checklists can be thought to encourage overly standardised reporting or ‘over‐obedience’, which can reduce the researchers' critical reflection and depth of qualitative research (Buus et al., 2025). Future work could incorporate a reporting framework, such as COREQ, while still aligning with a reflexive qualitative approach (Braun & Clarke, 2024).

### Clinical implications

The results have important clinical implications for improving access to psychological therapies for individuals who self‐harm. The results highlight the importance of collaborative, person‐centred early intervention, involving EbEs and focussing on understanding individuals' needs and reasons for self‐harm. Experiences of rejection from services and diagnostic labels given by professionals hindered further help‐seeking and access to support. This is contrary to NICE guidelines (2022), which state that, at the earliest opportunity, health professionals should inform people how to access support and that access should not be determined by diagnosis or co‐existing conditions. In line with research by Leather et al. (2020), who highlighted that healthcare professionals implement the NICE guidelines for self‐harm in less than 50% of encounters with individuals who self‐harm, further work is needed to ensure consistent adherence to these national guidelines.

The results indicated that EbE involvement should be a common feature of services. Peer support groups were also identified as a viable resource where individuals who self‐harm could openly discuss experiences in a non‐judgemental and accepting environment. In line with previous research, results highlighted that EbEs can be fundamental to the contribution of ideas within mental health services and can empower individuals to seek help for self‐harm (Rocelli et al., 2024; Veldmeijer et al., 2023). Further research with individuals with lived experience and clinicians working within the NHS is needed to explore how EbEs can become embedded in routine mental health services to ultimately improve service delivery and satisfaction.

Key areas of NPT help to identify how readily a new intervention, such as CAT (as evaluated in the RELATE trial), can be adopted and become routine practice in services (Murray et al., 2010). It is important to consider the aspects of NPT when designing and implementing a new therapy into services. For example, both stakeholder groups perceived a brief intervention as a useful intervention, especially in the early stages of self‐harm. Results suggested high levels of ‘buy‐in’ for developing more specific self‐harm‐related interventions amongst both individuals with lived experience and healthcare professionals. However, the sense of being powerless to make changes and overcome barriers within the system (i.e. long waiting times) should be considered. Stakeholders' levels of collective action and confidence in a new brief intervention could help overcome these barriers to support for people who self‐harm. The identified barriers to accessing self‐harm support and implementation outlined by both stakeholder groups should be considered and addressed before a new brief therapy is implemented within the NHS. Table 4 summarises how each theme relates to the core NPT constructs.

**TABLE 4:** Summary of how themes map onto NPT concepts.

Theme	NPT constructs	The construct within participants' accounts
1. Feeling powerless within the system	Coherence	Participants shared a clear understanding that existing care often does not meet the needs of people who self‐harm, consistent with participants describing long waiting times and being passed between services. Indicating a recognised need for change
	Collective action	Both groups described limited capacity to enact or sustain change within current service constraints (e.g. staffing, eligibility criteria, fragmented pathways)
	Reflexive monitoring	Stakeholders appraised the effects of current arrangements (e.g. disengagement, deterioration during waits)
2. Understanding the person behind self‐harm	Coherence	Participants emphasised that support should address the ‘why’ behind self‐harm. This highlighted that behaviour‐focussed approaches for self‐harm can ignore the underlying factors contributing to self‐harm
	Cognitive participation	There was visible ‘buy‐in’ to more person‐centred, relational approaches (including culturally sensitive work), reflecting willingness to engage with alternatives to behaviour‐focussed therapies
	Reflexive monitoring	Participants explained the difficulties of current practices (e.g. DBT/CBT as ‘sticking plasters’ for some) and outlined what ‘better’ support should involve (deeper exploration, reduced shame/stigma)
3. What could self‐harm support look like?	Cognitive participation	Both groups expressed readiness to consider new options (e.g. early, brief interventions) while acknowledging different preferences and needs
	Collective action	Practical considerations were outlined (timing, session length, planned endings, staff skills), indicating attention to what would make an intervention work in current settings
	Reflexive monitoring	Stakeholders weighed potential benefits and limits of brief therapy (e.g., useful early on vs. insufficient for complex needs), informing judgements about its suitability and likely impact

Abbreviation: NPT, normalisation process theory.

## CONCLUSIONS

These findings offer valuable insight into the experiences of individuals with lived experience of self‐harm and clinicians working with people who self‐harm. These highlight both shared perceptions of the barriers and facilitators to accessing care and some differences around the potential value of brief interventions. Both groups reported significant barriers to access, including a shared sense of powerlessness in addressing systemic and service‐level challenges such as long wait‐times and staff shortages. The findings of this study highlight a need for services to offer more specific self‐harm‐related interventions, which focus on understanding self‐harm and the person behind the behaviour and have a clear and well‐planned ending. In addition, services should include EbE involvement in training for clinicians, peer support, person‐centred care and provide rapid access at the earliest opportunity to prevent people who self‐harm from falling into the gaps between service tiers.

## AUTHOR CONTRIBUTIONS

**Tara Chauhan:** Writing – original draft; methodology; writing – review and editing; formal analysis; data curation; resources; conceptualization; investigation; funding acquisition; software; validation; visualization. **Kamelia Harris:** Supervision; formal analysis; writing – review and editing; conceptualization; methodology; investigation; resources; data curation; visualization; project administration. **Isabel Adeyemi:** Writing – review and editing; data curation; resources; formal analysis; project administration. **Cameron Latham:** Writing – review and editing; methodology; supervision; conceptualization. **Catherine Robinson:** Supervision; conceptualization. **Stephen Kellett:** Writing – review and editing. **Peter Taylor:** Conceptualization; funding acquisition; writing – review and editing; methodology; visualization; project administration; supervision; resources; data curation; formal analysis.

## FUNDING INFORMATION

This study/project is funded by the NIHR Research for Patient Benefit Programme (NIHR unique award identifier: NIHR203515). The views expressed are those of the author(s) and not necessarily those of the NIHR or the Department of Health and Social Care. The project sponsor is Greater Manchester Mental Health NHS Foundation Trust (Bury New Rd, Prestwich, Manchester M25 3BL; researchoffice@gmmh.nhs.uk). The project funder and sponsor did not have any role in the design or reporting of this protocol.

## CONFLICT OF INTEREST STATEMENT

The authors declare no conflicts of interest.

## Data Availability

The data that support the findings of this study are available from the corresponding author upon reasonable request.
